# Horrifying shift of a giant thrombus during coronary intervention

**DOI:** 10.1093/ehjcr/ytac177

**Published:** 2022-04-21

**Authors:** Kentaro Fukuda, Takehiro Funamizu, Hiroshi Tamura, Kikuo Isoda

**Affiliations:** Department of Cardiology, Juntendo University Nerima Hospital, 3-1-10, Takanodai, Nerima-ku, 177-8521 Tokyo, Japan

A 71-year-old female with acute myocardial infarction underwent diagnostic coronary angiography (DCA). Her ectatic left circumflex (LCx) coronary artery showed total thrombotic occlusion (see Supplementary material online, *Video S1*) (*Figure 1A* arrows). Intravascular ultrasound guidance over a wire in the left anterior descending (LAD) coronary artery was used to locate the LCx ostium, and manual thrombus aspiration followed by balloon angioplasty was done. A thrombus that had migrated into the guiding catheter was subsequently injected into the LAD (*Figure 1B* arrows) (see Supplementary material online, *Video S2*), resulting in ST-segment elevation in leads V1–V4 and subsequent complaints of chest pain. Further thrombus aspiration was performed, but Thrombolysis in Myocardial Infarction (TIMI) flow grade remained at 1. Intracoronary administration of 120 000 units of urokinase established TIMI 3 flow in the LAD. The patient was treated with aspirin 100 mg once daily, rivaroxaban 15 mg once daily, and a reduced dose (3.75 mg) of prasugrel once daily. Repeated DCA 1 week later showed complete resolution of the thrombus (*Figure 1C*) (see Supplementary material online, *Video S3*). Prasugrel was stopped, and the patient was maintained on long-term aspirin and rivaroxaban. The patient was discharged free of chest pain.

**Figure 1:**
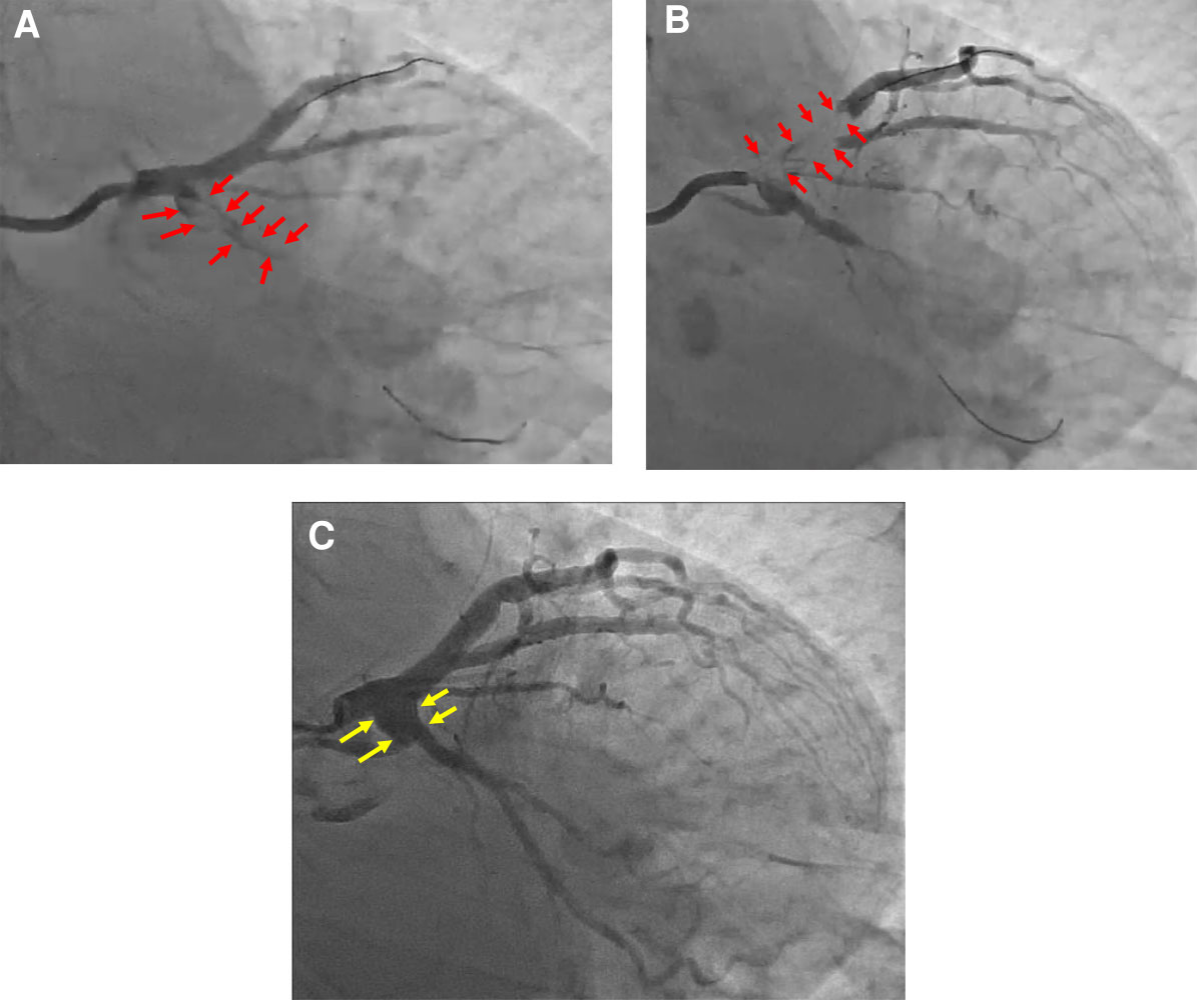
(*A*) Upon angiography, a large intracoronary thrombus burden was noticed in the left circumflex coronary artery (arrows). (*B*) A thrombus that had migrated into the guiding catheter was subsequently injected into the anterior descending artery (arrows). (*C*) A repeated angiogram after 1 week revealed the dissolution of the thrombus with optimal medical therapy and coronary artery ectasia (arrows).

Thrombus aspiration has been used in many patients with acute myocardial infarction in Japan because of its appealing effect on the reduction of thrombus burden and no-reflow phenomena.^1^ Our case suggested that careful flushing of the guide catheter must be done to avoid the prevention of a thrombus embolization after aspiration thrombectomy, as in a previous report.^2^ Furthermore, pressure damping was initially thought to be due to cardiogenic shock, and in hindsight, it should have triggered a further evaluation of the guide catheter for the presence of a thrombus.

Our patient had coronary artery ectasia (CAE) (Figure 1C arrows), and rivaroxaban was added as part of the pharmacological therapy, as in the case of CAE. The addition of this drug has been thought to reduce the recurrence of myocardial infarction,^3^ although more studies are required to support this practice.

## Supplementary Material

ytac177_Supplementary_Data
